# Rek-Surv: A lightweight deep survival model for plant infectious disease onset prediction

**DOI:** 10.1016/j.idm.2026.03.001

**Published:** 2026-03-04

**Authors:** Jinggui Xiao, Shan Hu, Xiaoling Deng, Yubin Lan, Meirong Xu, Fen Dai, Yuhe Li, Junchao You, Guangnan Zhang, Yongshun Liu

**Affiliations:** a College of Electronic Engineering (College of Artificial Intelligence), South China Agricultural University, Guangzhou, 510642, China; b National Center for International Collaboration Research on Precision Agricultural Aviation Pesticide Spraying Technology, Guangzhou, 510642, China; c Guangdong Engineering Technology Research Center of Smart Agriculture, Guangzhou, 510642, China; d Guangdong Province Key Laboratory of Microbial Signals and Disease Control, Citrus Huanglongbing Research Laboratory, South China Agricultural University, Guangzhou, 510642, China; e College of Mathematical, Physical and Computational Sciences (College of Climate Change and Artificial Intelligence), University of Reading, RG6 6UR, UK

**Keywords:** Deep survival analysis, Kolmogorov-Arnold networks, Residual mechanism, Agricultural disease prediction, Citrus Huanglongbing, Precision agriculture

## Abstract

Infectious disease outbreaks in crop systems pose a significant threat to global food security, particularly when early detection and intervention opportunities are missed. Predicting not just whether but when an infection will emerge is critical for effective disease management and control. In this context, survival analysis—a statistical framework for modeling time-to-event data—offers a natural and powerful solution. Widely adopted in epidemiology and clinical research, survival analysis can be adapted to plant disease surveillance for predicting the timing of infection onset at the host or population level.

However, deep survival models based on multilayer perceptrons (MLPs) often struggle with high-dimensional agricultural data, leading to issues such as overfitting, poor parameter efficiency, and limited interpretability. To address these challenges, we introduce a Kolmogorov–Arnold Network (KAN) architecture into the survival analysis context, leveraging its compact nonlinear function-approximation capabilities to improve predictive performance and efficiency. We present Rek-Surv, a lightweight deep survival model built on an Efficient-KAN backbone and augmented with residual connections and enhanced regularization. Rek-Surv is evaluated on five clinical benchmark datasets as well as a citrus Huanglongbing (HLB) plant disease dataset, demonstrating its generalizability across human and plant infectious disease contexts. On the HLB task, Rek-Surv achieves a high concordance index (C-index) of 0.962 with only 114 trainable parameters and millisecond-level inference speed. It outperforms existing survival models by providing more accurate outbreak onset predictions with a fraction of the model complexity. This efficiency makes Rek-Surv well-suited for real-time outbreak detection and early intervention, illustrating how advanced survival models can enable proactive infectious disease management in both agriculture and healthcare.

## 1 Introduction

### 1.1 Background and motivation

Plant disease epidemics pose a serious threat to global food security and sustainable agriculture. Crop diseases cause over $220 billion in annual losses worldwide, disproportionately affecting smallholder farmers who often lack timely access to interventions (Food and Agriculture Organization of the United Nations, 2023). For example, the 2020 locust infestation in East Africa devastated maize yields by up to 70%, and the ongoing spread of citrus Huanglongbing (HLB) – an infectious bacterial disease – has cut global orange production by roughly 20% since 2010 (Gottwald et al., 2020; World Bank, 2021). These crises highlight the urgent need for early outbreak detection systems capable of identifying emerging plant disease epidemics before irreversible damage occurs.

One promising approach for modeling the timing of such outbreaks is survival analysis, a statistical framework originally developed in medical research. Survival analysis models time-to-event data (e.g., time until a plant becomes infected) and can naturally accommodate right-censored observations, making it uniquely suited for long-term agricultural monitoring where the exact infection time for some subjects remains unknown.

### 1.2 Related work

Historically, the Cox proportional hazards model (Cox, 1972) has been the gold standard for survival analysis due to its interpretability. However, its reliance on restrictive linear assumptions often limits its performance in capturing the complex, non-linear dynamics of biological systems. With the development of AI, machine learning extensions like random forest survival analysis (Ishwaran et al., 2011) and semi-parametric Bayesian models (Fernandez et al., 2016) were introduced to handle higher-dimensional data.

In recent years, deep learning has revolutionized the field. Models such as DeepSurv (Katzman et al., 2018) leveraged Multi-Layer Perceptrons (MLPs) to relax the linear constraints of Cox models. Furthermore, sophisticated architectures like CNNs and Transformers have been successfully applied to process unstructured data, such as histological images and genomic features, for multimodal survival prediction (Lipkova et al., 2022; Jaume et al., 2024, pp. 11579–11590).

However, a critical gap remains in the agricultural domain. Most state-of-the-art deep survival models are “heavyweight,” designed for high-dimensional data and requiring massive computational resources. In contrast, agricultural monitoring typically relies on low-dimensional tabular sensor data (e.g., temperature, humidity) and resource-constrained edge devices. Implementing heavy CNN or Transformer-based models on solar-powered sensors or low-cost UAVs is often impractical.

Recently, the Kolmogorov-Arnold Network (KAN) has emerged as a promising lightweight alternative due to its compact and highly nonlinear function approximation capabilities (Liu et al., 2025). Unlike MLPs, KANs use learnable activation functions on edges, offering greater efficiency. Nevertheless, baseline KAN models such as CoxKAN still face stability issues during training and remain prone to overfitting on smaller datasets (Knottenbelt et al., 2025).

### 1.3 Contributions of this work

To address these limitations, we propose Rek-Surv, a lightweight deep survival model based on the Efficient KAN architecture (Blealtan, 2024). Rek-Surv introduces two key innovations: (1) the inclusion of dual regularization terms in the negative log-likelihood loss function to mitigate overfitting, and (2) the incorporation of residual connections within the Kolmogorov-Arnold Network (KAN) framework for survival analysis tasks, to stabilize the training process. These design choices collectively enhance both predictive performance and inference efficiency, enabling practical deployment in real-time early-warning systems for agriculture and healthcare.

Rek-Surv is evaluated on five clinical datasets and a proprietary Huanglongbing (HLB) citrus disease dataset. It achieves a concordance index (C-index) of 0.962 on the HLB task, using only 114 trainable parameters and an inference latency of 0.0066 ms per sample—making it suitable for edge-based applications like UAV monitoring and in-orchard disease detection.

The key contributions of this work are:

1. The inclusion of dual regularization terms in the negative log-likelihood loss function to mitigate overfitting.
2. The incorporation of residual connections within the Kolmogorov-Arnold Network (KAN) framework for survival analysis tasks, to stabilize the training process.
3. Validation of Rek-Surv across six datasets, including a real-world agricultural task (HLB forecasting), demonstrating superior accuracy and deployment efficiency.

The remainder of this paper is structured as follows: Section 2 delineates the architecture and innovations of the proposed Rek-Surv model. Section 3 outlines the experimental design, including detailed descriptions of the evaluation metrics, comparative analyses, and empirical results. Section 4 presents a comprehensive discussion of the findings and their broader implications, while Section 5 offers concluding remarks and highlights avenues for future research.

## 2 Proposed Rek-Surv

Rek-Surv is a lightweight proportional hazards model built upon the Kolmogorov–Arnold Network (KAN) paradigm, designed to improve robustness and parameter efficiency for deep survival analysis. Our model is developed as an enhanced variant of Efficient-KAN, targeting two recurring issues observed when applying expressive spline-parameterized networks to censored survival data: optimization instability and overfitting under limited event information. Rek-Surv addresses these issues by integrating (i) static residual connections (He et al., 2016) to stabilize training dynamics and facilitate feature reuse, and (ii) Elastic Net regularization (Zou & Hastie, 2005) to improve generalization while retaining fast inference.

**Fig. 1:**
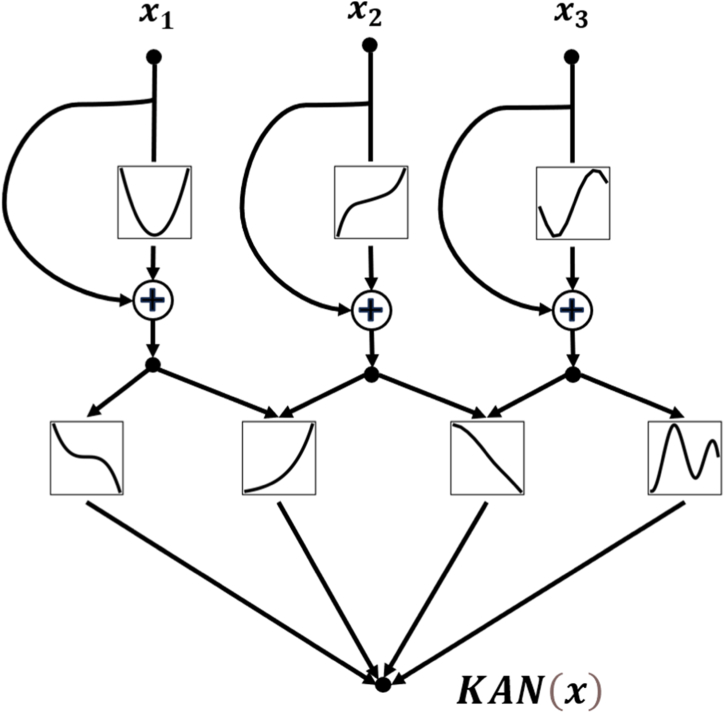
Visualization of Rek-Surv Network with shape [3,3,1] - the nodes are connected by learnable activation functions.

**Fig. 2:**
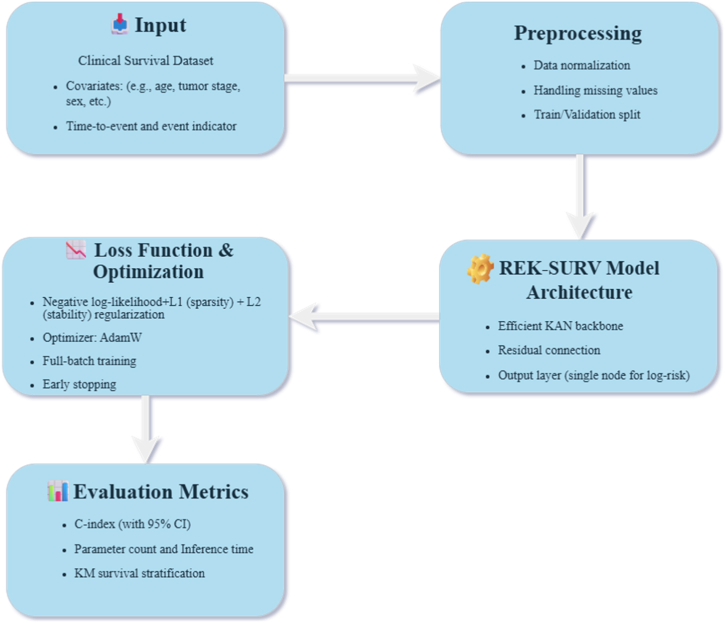
Workflow of the proposed Rek-Surv deep survival analysis methodology.

Following the proportional hazards formulation, Rek-Surv estimates the log-relative risk as

(1) $$\eta \left(x\right)=f_{\theta }\left(x\right)$$

where $f_{\theta }\left(\cdot \right)$ is implemented by an Efficient-KAN backbone augmented with residual pathways (Fig. 1). Fig. 2 provides an overview of the end-to-end pipeline; experimental protocols and selection criteria are detailed in Section 3.

### 2.1 Residual-enhanced KAN architecture

KANs achieve high functional expressiveness by learning spline-parameterized transformations on edges. While this property can improve parameter efficiency, stacking spline-based transformations may exacerbate optimization difficulty and gradient instability, especially in survival settings with censoring and heterogeneous covariates. Residual connections are a simple and widely adopted mechanism to ease optimization by providing an identity shortcut for information and gradient flow (He et al., 2016). Motivated by this principle and informed by the ResidualKAN implementation details (Withray, 2024), Rek-Surv augments Efficient-KAN with static residual pathways. Specifically, for the l-th layer, we reformulate the output as

(2) $$h_{l}=KAN_{l}\left(h_{l-1}\right)+h_{l-1}$$

This formulation preserves the expressive functional mapping while maintaining a direct feature reuse path. The benefit of the residual component is quantified in the ablation and robustness analyses in Sections 3.2, 3.5.

### 2.2 Loss function and optimization

Rek-Surv is trained with the Cox partial log-likelihood augmented by elastic-net–style regularization(Eq. (3)):

(3) $$L\left(\theta \right)=L_{Cox}\left(\theta \right)+\lambda_{1}{\left\|\theta \right\|}_{1}+\lambda_{2}{\left\|\theta \right\|}_{2}^{2}$$

where $\lambda_{1}$ promotes sparsity and $\lambda_{2}$ controls shrinkage. This design aims to suppress spurious spline components and reduce variance in high-dimensional settings, mitigating overfitting that can arise from KAN's strong nonlinear fitting capacity.

To optimize this objective, we combine AdamW-style decoupled weight decay with a proximal update for the non-smooth L1 term(Loshchilov & Hutter, 2019). Specifically, the L1 component is applied via element-wise soft-thresholding after the gradient-based update:

(4) $$\theta \leftarrow S_{\eta \lambda_{1}}\left(\theta \right),S_{\tau }\left(\theta \right)=\text{sign}\left(\theta \right)\cdot \max\left(\left|\theta \right|-\tau ,0\right)$$

where η denotes the learning rate. The L2 component is implemented through AdamW's decoupled weight decay, which provides an L2-like shrinkage effect. This method controls model complexity without the need for an additional explicit L2 penalty term in the loss function, ensuring both robustness and the lightweight nature of Efficient-KAN.

### 2.3 Comparative summary with existing methods

Table 1 summarizes representative survival baselines spanning classical proportional hazards models and neural survival approaches. CoxPH is highly interpretable but limited by linear risk assumptions, whereas DeepSurv improves flexibility via MLPs at the cost of increased parameters and slower inference. CoxKAN is a closely related KAN-based survival model that leverages spline-parameterized transformations for compact modeling; however, its performance can be sensitive to capacity and regularization choices and may exhibit training instability or overfitting under heavy censoring or small-to-moderate event regimes.

**Table 1:** Comparison of existing deep survival methods.

Method	Parametric Type	Advantages	Limitations
CoxPH	Semi-parametric	Interpretable, efficient	Linear assumption, limited scalability
DeepSurv	MLP-based	Captures nonlinearity	High parameters, slow, black-box
CoxKAN	KAN-based	Parameter-efficient, partially interpretable	Overfitting, unstable
Rek-Surv	KAN + Residual + Reg.	Lightweight, stable, fast inference	Limited multimodal input support

Rek-Surv differs from CoxKAN in that it is designed as a deployment-oriented KAN survival model with an explicit focus on stability and efficiency. Rek-Surv builds on an Efficient-KAN backbone and incorporates static residual pathways to improve optimization behavior in KAN-based survival learning, especially under censoring, which we further quantify via ablation and robustness experiments in Section 3. In addition, it adopts Elastic Net regularization that combines sparsity and shrinkage for complexity control. We evaluate these design choices with ablation and robustness analyses and report both discrimination and deployability metrics in the experimental section.

## 3 Experimental setup and results

We evaluate Rek-Surv on five publicly available clinical survival benchmarks—WHAS (Hosmer et al., 2008), METABRIC (Curtis et al., 2012), RGBSG (Schumacher et al., 1994), SUPPORT (Knaus et al., 1995), and NWTCO (Green et al., 1995)—covering diverse cohort sizes, feature dimensions, and censoring patterns. We follow the standard preprocessing and evaluation protocols used in prior survival modeling studies for each dataset. In addition, we validate Rek-Surv on a real-world citrus Huanglongbing (HLB) early-warning dataset collected from commercial orchards over a nine-month observation window, where the event is the first confirmed detection of infection and the target is time-to-detection under right censoring (Section 3.4).

### 3.1 Evaluation metrics

We assess models using the concordance index (C-index) (Harrell et al., 1996), parameter count, and inference speed (ms/sample). The C-index measures the model's accuracy in ranking survival times, while parameter count and inference speed are critical metrics for real-time deployment, especially for edge-based applications in agriculture.

### 3.2 Ablation study

In our ablation study, we further analyze where Rek-Surv's performance gains come from. Rek-Surv is built on an Efficient-KAN backbone and introduces residual connections together with elastic-net–style regularization. We therefore conduct ablation experiments to quantify the contributions of these design choices under a controlled training protocol.

We first report the overall gain of the full Rek-Surv design over the Efficient-KAN backbone in Table 2, where the combination of residual stabilization and regularization improves discrimination while mitigating overfitting. To provide a finer-grained attribution, we additionally perform a component-wise ablation using a full 2^3^ factorial design that toggles three binary factors: residual connections (Res), L1-induced sparsity (L1), and L2-like shrinkage (L2). The complete eight-variant results are reported in Appendix Table 7, and the corresponding factorial effect estimates (main and interaction effects) are summarized in Table 3.

**Table 2:** Clinical datasets: C-Index (95% Confidence Interval). Highest C-Index in bold.

Dataset	baseline Efficient KAN	Rek-Surv
WHAS	0.9155 (0.9148,0.9162)	0.9232 (0.9225,0.9238)
METABRIC	0.6306 (0.6293, 0.6319)	0.6571 (0.6558,0.6585)
RGBSG	0.6882 (0.6873, 0.6891)	0.6920 (0.6911,0.6930)
SUPPORT	0.6256 (0.6251, 0.6262)	0.6292 (0.6287,0.6298)
NWTCO	0.7003 (0.6986, 0.7019)	0.7233 (0.7217,0.7248)

**Table 3:** Main effects (ΔC-index, 95% bootstrap CI) from the $2^{3}$ factorial ablation on METABRIC.

Dataset	Res (ΔC-index)	L1 (ΔC-index)	L2 (ΔC-index)	Full – Baseline (ΔC-index)
METABRIC	0.01584 (0.0104,0.0214)	−0.0014 (−0.0066, 0.0027)	0.0015 (−0.0040,0.0055)	0.01505 (0.0102,0.0210)

Across the clinical benchmarks, the overall comparison in Table 2 indicates that the full Rek-Surv design improves discrimination while mitigating overfitting. To attribute these gains more precisely, we report a factorial ablation on METABRIC as a representative benchmark (Table 3), with the full eight-variant results provided in Appendix Table 7. The results support an interpretation consistent with survival learning dynamics: residual pathways stabilize optimization for spline-composed transformations, sparsity suppresses weak or noisy components, and shrinkage controls variance but may become detrimental when event information is scarce. We further examine robustness to key hyperparameters in Section 3.5.

### 3.3 Evaluation on real-world clinical datasets

To evaluate Rek-Surv's practical application, we compared its performance with CoxPH, DeepSurv, and CoxKAN on five clinical datasets (Table 4). In addition to the concordance index (C-index), we introduced model parameter count and inference speed as key efficiency metrics. Confidence intervals were obtained by bootstrapping the test set, and performance differences were considered statistically significant if the intervals did not overlap. While model compression and efficiency have been explored in other fields, few studies in deep survival analysis have systematically addressed both predictive accuracy and computational efficiency (Wiegrebe et al., 2024). Our evaluation aims to fill this gap, particularly for resource-constrained scenarios such as edge computing and point-of-care diagnostics.

**Table 4:** Clinical datasets: C-Index (95% Confidence Interval). Highest C-Index in bold.

Dataset	CoxPH	DeepSurv	CoxKAN	Rek-Surv
WHAS	0.816025 (0.813,0.819)	0.866723 (0.863,0.870)	0.854326 (0.854,0.856)	0.9232 (0.9225,0.9238)
METABRIC	0.632363 (0.628,0.637)	0.643375 (0.639,0.647)	0.649618 (0.644,0.651)	0.6571 (0.6558,0.6585)
RGBSG	0.656291 (0.655,0.662)	0.668402 (0.665,0.671)	0.682796 (0.678,0.684)	0.6920 (0.6911,0.6930)
SUPPORT	0.583074 (0.581,0.585)	0.618308 (0.616,0.620)	0.624485 (0.622,0.625)	0.6292 (0.6287,0.6298)
NWTCO	0.698347 (0.693, 0.703)	0.698300 (0.692, 0.703)	0.722225 (0.715,0.725)	0.7233 (0.7217,0.7248)

As shown in Table 3, Rek-Surv achieves the highest C-index across all five clinical datasets. Performance differences were considered statistically significant when the 95% confidence intervals (CIs) did not overlap. Based on this criterion, Rek-Surv shows significant improvements over all baseline models on WHAS, METABRIC, RGBSG, and SUPPORT. On the NWTCO dataset, the CI of Rek-Surv slightly overlaps with that of CoxKAN, indicating a marginal but not statistically significant difference. This may be due to both Rek-Surv and CoxKAN using the KAN architecture, which provides strong nonlinear approximation capabilities, leading to similar high performance on datasets with relatively clean structures, like NWTCO.

As shown in Table 5, Rek-Surv achieves competitive discrimination while remaining efficient on most benchmarks. For WHAS, we selected a slightly higher-capacity configuration (larger grid size and deeper setting) based on validation performance, which results in a modest increase in parameter count. This setting yields the best C-index on WHAS (0.9232) while retaining fast inference, indicating that Rek-Surv can trade a small amount of capacity for improved ranking performance when the dataset supports it.

**Table 5:** Model Parameter Counts and Inference Speeds on Various Clinical datasets. Best results are in bold (lower parameters and faster inference).

Dataset	Model	Parameter Count	Inference Speed(ms)
WHAS	DeepSurv	50561	34.816
	CoxKAN	731	21.059
	Rek-Surv	51408	16.657
METABRIC	DeepSurv	17921	19.813
	CoxKAN	109	6.523
	Rek-Surv	**60**	**2.246**
RGBSG	DeepSurv	13057	34.762
	CoxKAN	195	17.007
	Rek-Surv	**96**	**3.480**
SUPPORT	DeepSurv	35073	220.840
	CoxKAN	544	42.520
	Rek-Surv	**135**	**7.853**
NWTCO	DeepSurv	253	114.463
	CoxKAN	496	16.142
	Rek-Surv	**54**	**1.376**

### 3.4 Early warning of citrus Huanglongbing using Rek-Surv

To evaluate Rek-Surv's ability to perform early and accurate prediction of real-world crop disease outbreaks, we conducted an experiment on a time-to-event prediction task for citrus Huanglongbing (HLB). The objective is to predict, based on tree-specific and environmental features, when a healthy citrus tree will first test positive for HLB via qPCR. Such early prediction enables pre-symptomatic intervention and targeted pesticide application, thus directly supporting orchard-level disease management.

The proprietary dataset was collected from a citrus orchard in Huangtian Town, Sihui City, Guangdong Province, China, from June 2024 to February 2025. Each tree was monitored monthly, resulting in 764 follow-up records. The event label was defined as the first month when the tree's qPCR cycle threshold (CT) value exceeded a diagnostic threshold, indicating molecular confirmation of HLB infection.

The dataset comprises 18 input features, including both static variables (e.g., tree age, vigor, canopy dimensions, cultivar type, and presence of infected trees within a 2-m radius) and dynamic environmental and soil measurements (e.g., air temperature, humidity, rainfall, wind speed, light intensity, soil temperature, moisture, and electrical conductivity), collected hourly via a sensor network, and subsequently aggregated into monthly averages to align with the disease inspection frequency. Monthly, leaf samples were collected for each tree, and CT (cycle threshold) values were measured in the laboratory using qPCR to determine HLB infection status. The time-to-event label was defined as the first month in which a tree's CT value exceeded a predefined diagnostic threshold.

All numerical features were standardized to zero mean and unit variance. Categorical variables, such as cultivar type and presence of neighboring infected trees, were encoded using one-hot encoding.

Table 6 reports performance on the HLB early-warning task. Rek-Surv achieves a C-index of 0.9616 (95% CI: 0.9605–0.9621), outperforming DeepSurv and CoxKAN while remaining highly efficient (114 parameters; 0.0066 ms/sample). We attribute the strong performance on HLB to the interaction between dataset characteristics and model inductive biases: the task exhibits substantial right censoring and limited effective event information, under which optimization can be noisy and overfitting becomes more likely for expressive non-linear models. Residual stabilization improves gradient flow when fitting spline-composed transformations, and elastic-net–motivated complexity control suppresses spurious components and reduces variance, yielding a favorable accuracy–latency trade-off for latency-critical orchard monitoring.

**Table 6:** Comparative Performance and Inference Efficiency on the HLB Dataset. Highest C-Index in bold.

Model	C-index (95% CI)	Parameter Count	Inference Speed(ms)
DeepSurv	0.8971 (0.8943,0.8979)	1191	0.0090
CoxKAN	0.9218 (0.916,0.920)	230	0.1180
Rek-Surv	**0.9616 (0.9605,0.9621)**	**114**	**0.0066**

**Fig. 3:**
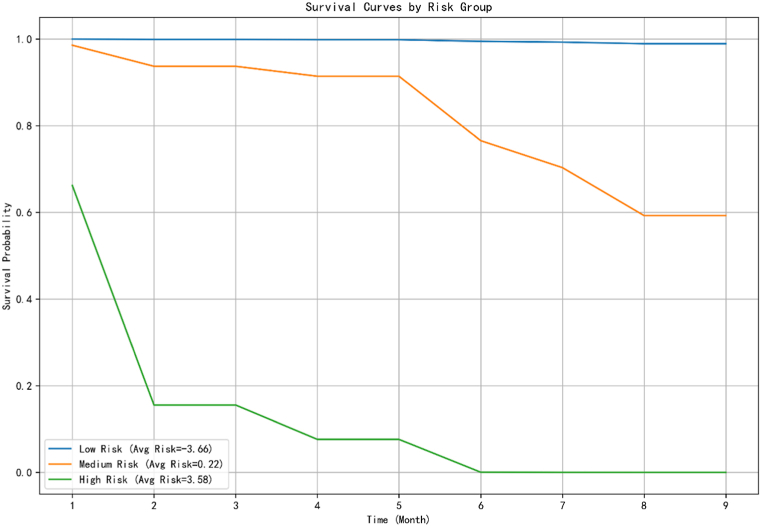
Risk-stratified survival curves for citrus trees based on Rek-Surv predictions.

To further assess Rek-Surv's utility for risk stratification and intervention planning, we grouped trees into low-, medium-, and high-risk categories based on their predicted log-risk scores. Fig. 3 shows the Kaplan–Meier survival curves for each group.

Notably, the survival probability for the high-risk group declines sharply during the first month—coinciding with the early summer period when vector activity of *Diaphorina citri* peaks—demonstrating both the biological relevance and real-time applicability of Rek-Surv's predictions.

Compared with molecular diagnostics like qPCR, which are accurate but delayed and infrastructure-intensive, Rek-Surv provides real-time, in-field risk estimation using only sensor-derived features and lightweight computation. This enables proactive monitoring and early intervention without dependence on lab-based testing, supporting the deployment of autonomous early-warning systems in smart orchards.

### 3.5 Hyperparameter sensitivity and robustness

**Fig. 4:**
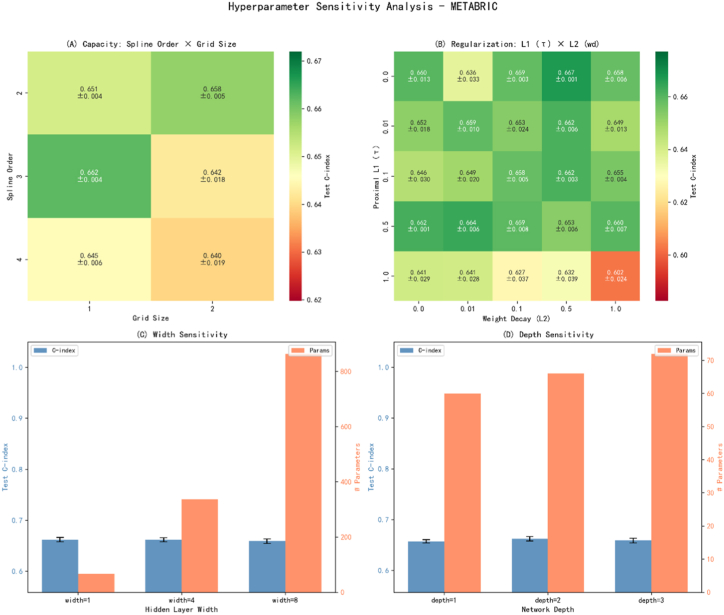
Hyperparameter sensitivity of Rek-Surv on METABRIC (mean ± std over 3 seeds).

Given practical concerns that KAN-based survival models can be sensitive to capacity and regularization, we perform a controlled robustness study by perturbing key hyperparameters around the selected operating point. Specifically, we vary regularization strength (L1 and L2-type shrinkage) and model capacity (depth, spline order, and grid size), and summarize the resulting performance landscapes in Fig. 4.

On METABRIC, we observe a stable operating region where performance remains competitive under moderate perturbations, with regularization strength exerting the clearest influence within the scanned range. In contrast, capacity-related parameters (depth, spline order, and grid size) mainly govern an explicit accuracy–efficiency trade-off, enabling Rek-Surv to maintain strong discrimination with low latency under resource constraints.

## 4 Discussion

Rek-Surv is motivated by a practical observation in censored survival learning: when effective event information is limited and censoring is substantial, highly flexible function-parameterized models can become sensitive to optimization and regularization, leading to unstable generalization. Within this regime, residual pathways offer a structurally simple way to stabilize the learning of spline-composed transformations, while elastic-net regularization provides complementary control of complexity by combining sparsity and shrinkage. Conceptually, this combination is well aligned with deployment-oriented survival modeling, where the goal is not only high discrimination but also a reliable operating region under realistic data constraints.

### 4.1 Limitations and future directions

A limitation of the current HLB study is the relatively short observation horizon (nine months) and the fact that data are collected under a fixed monitoring protocol. In longer-term agricultural deployments, covariate distributions and risk mechanisms may drift due to seasonal patterns, management changes, and evolving pathogen pressure, which can challenge the stationarity assumptions implicit in standard proportional-hazards modeling. A natural extension is to incorporate time-dependent covariates or time-varying effects (e.g., piecewise or landmarked modeling) to better capture long-horizon dynamics.

From a scalability perspective, Rek-Surv's lightweight design is compatible with continuous or periodic model updates as new monitoring data arrive. In practice, this could be implemented via scheduled re-training with warm starts, sliding-window updates to emphasize recent seasons, or drift-triggered updates when performance indicators shift. Regularization and sparsity can further help stabilize incremental fitting by discouraging spurious fluctuations, although careful evaluation is needed to avoid catastrophic forgetting and to account for delayed or uncertain event labels in field settings. Future work will include multi-orchard and multi-season validation and systematic study of continual learning protocols for robust long-term deployment.

## 5 Conclusion

In this study, we introduced Rek-Surv, a lightweight deep survival model based on the Efficient Kolmogorov-Arnold Network (KAN). By incorporating residual connections and Elastic Net regularization, Rek-Surv addresses key challenges such as overfitting and low parameter efficiency, making it well-suited for real-time outbreak prediction in both healthcare and agriculture. Its efficient design allows deployment in resource-constrained environments, such as mobile health systems and smart orchards, where computational resources are limited. Moreover, Rek-Surv's ability to generalize across both human and plant infectious diseases underscores its broad potential for disease outbreak prediction.

## CRediT authorship contribution statement

**Jinggui Xiao:** Writing – review & editing, Writing – original draft, Visualization, Validation, Supervision, Software, Resources, Project administration, Methodology, Investigation, Formal analysis, Data curation, Conceptualization. **Shan Hu:** Writing – review & editing, Writing – original draft, Validation, Supervision, Software, Methodology, Investigation, Formal analysis, Data curation. **Xiaoling Deng:** Writing – review & editing, Writing – original draft, Validation, Supervision, Resources, Project administration, Methodology, Investigation, Funding acquisition, Formal analysis, Data curation, Conceptualization. **Yubin Lan:** Writing – review & editing, Supervision, Project administration, Methodology, Investigation, Formal analysis, Data curation, Conceptualization. **Meirong Xu:** Supervision, Resources, Project administration, Methodology, Investigation, Formal analysis, Data curation, Conceptualization. **Fen Dai:** Writing – review & editing, Supervision, Resources, Project administration, Methodology, Investigation, Formal analysis, Data curation, Conceptualization. **Yuhe Li:** Writing – original draft, Validation, Resources, Methodology, Investigation, Formal analysis, Data curation, Conceptualization. **Junchao You:** Writing – original draft, Supervision, Software, Resources, Investigation, Formal analysis, Data curation, Conceptualization. **Guangnan Zhang:** Writing – original draft, Resources, Project administration, Methodology, Investigation, Formal analysis, Data curation, Conceptualization. **Yongshun Liu:** Writing – original draft, Methodology, Investigation, Formal analysis, Data curation, Conceptualization.

## Code availability

The source code for Rek-Surv, including the implementation of the Residual KAN layers and the survival analysis training pipeline, is available on GitHub at [https://github.com/jesseguigui/ReK-Surv].

## Declaration of competing interest

The authors declare that they have no known competing financial interests or personal relationships that could have appeared to influence the work reported in this paper.

## Data Availability

The real-world medical datasets generated and/or analysed during the current study are available in the GitHub repository: [Rek-Surv Datasets], [https://github.com/jesseguigui/rek-surv-datasets/tree/main]. The real-world agricultural dataset of healthy citrus trees will be made available on request.
