# Supplementary material for: Rek-Surv: A lightweight deep survival model for plant infectious disease onset prediction
Source: Infect Dis Model. 2026 Mar 4;11(4):1270–9. doi: 10.1016/j.idm.2026.03.001 (PMC13196313; doi:10.1016/j.idm.2026.03.001)
Supplement: Multimedia component 1 [file mmc1.docx]

**Supplementary Materials: Hyperparameter Configurations**

**Appendix A**

Table 4 Hyperparameters of Rek-Surv

| Hyperparameter | WHAS | METABRIC | RGBSG | SUPPORT | NWTCO |
| --- | --- | --- | --- | --- | --- |
| KAN Shape | [6,24,1] | [9,1,1] | [7,1,1] | [14,1,1] | [13,1,1] |
| Base Activation | ReLu | ReLu | ReLu | ReLu | ReLu |
| Learning Rate | 0.001 | 0.001 | 0.001 | 0.001 | 0.001 |
| Weight Decay | 0.0001 | 0.0001 | 0.0001 | 0.0001 | 0.0001 |
| L1 Regularization ($\lambda_{1}$) | 0.1 | 0.1 | 0.1 | 0.1 | 0.1 |
| L2 Regularization ($\lambda_{2}$) | 0.5 | 0.5 | 0.5 | 0.5 | 0.5 |
| Epoch | 491 | 490 | 280 | 500 | 165 |
| Grid Eps | 0.01 | 0.01 | 0.01 | 0.01 | 0.1 |
| Grid Range | [-5, 5] | [-1, 1] | [-1, 1] | [-1, 1] | [-1, 1] |
| Grid Size | 300 | 1 | 7 | 3 | 4 |
| Scale Base | 2.0 | 1.0 | 1.5 | 1.0 | 2.0 |
| Scale Noise | 0.1 | 1 | 0.5 | 0.1 | 0.1 |
| Scale Spline | 2.0 | 1 | 1.5 | 1.0 | 1.5 |
| Spline Order | 4 | 3 | 3 | 1 | 3 |

Table 5 Hyperparameters of DeepSurv.

| Hyperparameter | NWTCO |
| --- | --- |
| Shape  Early Stopping | [6,9,1]  False |
| Epochs | 135 |
| Learning Rate | 0.008 |
| Batch Norm | True |
| Dropout  Weight Decay(L2) | 0.12  6.6e-8 |

Table 6 Hyperparameters of CoxKAN.

| Hyperparameter | WHAS | METABRIC | RGBSG | SUPPORT | NWTCO |
| --- | --- | --- | --- | --- | --- |
| KAN Shape | [6,5,5,1] | [9,1] | [7,2,1] | [14,3,1] | [6,5,1] |
| Learning Rate | 0.01 | 0.09 | 0.0076 | 0.015 | 0.002 |
| Early Stopping | True | True | True | True | False |
| Steps | (300) | (300) | (300) | (300) | 147 |
| Prune threshold | 0.047 | 0.035 | 0.045 | 0.00007 | 0.02 |
| Grid Intervals | 3 | 3 | 3 | 3 | 5 |
| Base fn | silu | silu | silu | linear | linear |
| Spline noise | 0.085 | 0.1 | 0.09 | 0.11 | 0.15 |
| Base noise | 0.16 | 0.03 | 0.18 | 0.05 | 0.16 |
| Reg | 0.003 | 0.003 | 0.0007 | 0.005 | 0.002 |
| Entropy Reg | 1 | 0 | 3 | 2 | 2 |
| Coeffcient Reg | 2 | 4 | 2 | 4 | 2 |

Table 7. Factorial Abalation Results for METABRIC (Full 8-Variant Design)

| Effect Type | Component / Interaction | Mean Effect | 95% CI | Statistical Significance |
| --- | --- | --- | --- | --- |
| Main Effect | Res | +0.0158 | [0.0104, 0.0214] | Yes (p < 0.05) |
|  | L1 Regularization | -0.0014 | [-0.0066, 0.0027] | No |
|  | L2 Regularization | +0.0015 | [-0.0040, 0.0055] | No |
| Interaction | Res x L1 | -0.0035 | [-0.0074, 0.0013] | No |
|  | Res x L2 | +0.0031 | [-0.0022, 0.0084] | No |
|  | L1 x L2 | +0.0036 | [-0.0016, 0.0113] | No |
| Overall | Total Improvement | +0.0151 | [0.0102, 0.0210] | Yes (p < 0.05) |
